# Agreement and Reliability of the G-Force System: Force Plate and Load Cell for the Isometric Mid-Thigh Pull in Physically Active Adults: A Repeated-Measures Method-Comparison Study

**DOI:** 10.3390/s26134178

**Published:** 2026-07-02

**Authors:** Héctor Fuentes-Barría, Víctor Garrido-Osorio, Raúl Aguilera-Eguía, Ángel Roco-Videla, Marcela Caviedes-Olmos, Lisse Angarita-Davila, Cherie Flores-Fernandez, Jorge Leschot-Gatica, Sebastián Sanhueza-González, Alejandro Pérez Castilla

**Affiliations:** 1 Centro de Investigación en Medicina de Altura (CEIMA), Universidad Arturo Prat, Iquique 1110939, Chile; hefuentes_@unap.cl; 2 Escuela de Ciencias de la Actividad Física, Facultad de Salud y Ciencias Sociales, Universidad de las Américas, Concepcion 4030000, Chile; vgarrido@fitbike.cl (V.G.-O.); jleschotg@gmail.com (J.L.-G.); 3 Departamento de Salud Pública, Facultad de Medicina, Universidad Católica de la Santísima Concepción, Concepcion 3349001, Chile; raguilerae@ucsc.cl; 4 Dirección de Desarrollo y Postgrado, Universidad Autónoma de Chile, Santiago 7500912, Chile; angel.roco@uautonoma.cl; 5 Facultad de Salud y Ciencias Sociales, Universidad de las Americas, Providencia, Santiago 7500975, Chile; 6 Escuela de Nutricion y Dietetica, Facultad de Medicina, Universidad Andres Bello, Concepcion 4030000, Chile; lisse.angarita@unab.cl; 7 Departamento de Gestión de la Información, Universidad Tecnológica Metropolitana, Santiago 7550000, Chile; cflores@utem.cl; 8 Escuela de Entrenamiento Deportivo, Facultad de Educación y Ciencias Sociales, Universidad Andres Bello, Concepcion 3349001, Chile; sebastianshzgonz@gmail.com; 9 Departamento de Educación, Facultad de Ciencias de la Educación, Universidad de Almería, 04120 Almería, Spain; alexperez@ual.es

**Keywords:** validation study, equipment and supplies, reproducibility of results, muscle strength, isometric contraction

## Abstract

**Objectives**: We aimed to compare the agreement and reliability of the G-Force system (force plate and load cell) for the mid-thigh isometric pull test in physically active adults. **Methods:** Eighteen participants (age: 27.6 years; BMI: 27.8 kg/m^2^) performed three maximal IMTP trials under standardized conditions. Force-time data were collected simultaneously from both systems. Peak force and rate of force development (RFD) were analyzed. Agreement was assessed using intraclass correlation coefficients (ICC) and Bland–Altman analysis. Paired *t*-tests and Cohen’s d evaluated systematic differences. Linear regression analyses were conducted to assess proportional bias. **Results:** Peak force showed excellent agreement (ICC = 0.999; 95% CI: 0.998–1.000) but with a positive fixed bias (84.48 N) and narrow limits of agreement (42.10 to 126.90 N), indicating consistently higher values from the force plate. A statistically significant difference was observed (*p* < 0.001; d = 3.90). Although the standardized effect size was large, the absolute bias between devices was relatively small (84.48 N; ~6% of mean peak force). The elevated d value reflects the low variability of the inter-system differences rather than a substantial absolute discrepancy. Accordingly, the results indicate a consistent systematic bias that may affect direct interchangeability of absolute values despite the excellent agreement observed between systems. No proportional bias was detected (*p* = 0.159). In contrast, RFD max showed lower agreement (ICC = 0.887; 95% CI: 0.699–0.958), with a negative bias (−1361 N/s) and wide limits of agreement (−6168 to 3445 N/s). Differences were significant (*p* = 0.031; d = 0.55), with no proportional bias (*p* = 0.268). **Conclusions:** The G-Force system demonstrates acceptable agreement for peak force but cannot be considered interchangeable with force plate measurements due to the presence of substantial systematic bias. In contrast, agreement for RFD is reduced, indicating greater sensitivity of early-phase force-time measures to methodological differences between systems.

## 1. Introduction

Muscular strength plays a crucial role in human performance, particularly in sports, injury prevention, and rehabilitation contexts, where it is strongly associated with functional capacity and long-term physical health outcomes [1,2,3]. Among the different approaches used to assess strength, maximal isometric force production has gained increasing attention due to its strong relationship with dynamic athletic tasks such as sprinting, jumping, and rapid changes in direction [4,5]. In this context, the isometric mid-thigh pull (IMTP) has emerged as a widely adopted assessment tool in applied sports science, as it allows for the evaluation of maximal force output under highly controlled and standardized conditions [6,7]. Beyond peak force, the IMTP also enables the analysis of rate of force development (RFD), which provides additional insight into neuromuscular function and explosive performance capacity.

Force-time characteristics derived from the IMTP, particularly peak force and early-phase RFD, are considered key indicators of neuromuscular performance and functional capacity [7,8]. However, the accuracy and interpretability of these variables are highly dependent on methodological factors, including sampling frequency, signal filtering, and force onset detection procedures [9,10]. Small variations in these processing decisions can lead to meaningful differences in derived outcomes, especially for time-dependent variables such as RFD, which are inherently more sensitive to noise and signal instability. Consequently, ensuring measurement consistency and evaluating agreement between different acquisition systems is essential when interpreting force-time data in both research and applied settings.

Force plates are widely considered the reference standard for IMTP testing due to their high sampling frequencies, validated accuracy, and extensive use in biomechanical research for ground reaction force assessment [11]. Nevertheless, their high cost and limited portability often restrict their use in field-based environments, particularly in applied sports and clinical settings [12]. These practical limitations have stimulated growing interest in portable and lower-cost alternatives capable of providing accessible force-time assessment in applied settings. This limitation has driven the development of more affordable alternatives, including load cell–based systems and custom-built devices, which aim to provide comparable force measurement capabilities in more accessible formats [13].

Recent evidence suggests that low-cost force measurement systems can achieve acceptable levels of reliability and agreement when compared with force plates, particularly for maximal isometric strength assessments [14]. In parallel, advances in sensor technology and signal processing have improved the feasibility of using load cell configurations for capturing force-time data in applied contexts [15]. Accordingly, custom-built systems such as the G-Force device have emerged as practical alternatives for field-based neuromuscular assessment due to their reduced cost and portability. However, despite these developments, evidence regarding the reliability and concurrent agreement of portable low-cost systems with force plate-derived measurements during IMTP testing remains limited, particularly under simultaneous acquisition conditions that allow direct comparison of force-time outputs [7]. Importantly, this comparison is framed as an inter-device agreement analysis rather than an assessment of absolute validity, as both systems are evaluated relative to each other rather than against a laboratory-grade criterion reference.

Therefore, this study aimed to analyze the reliability of the G-Force system and examine its agreement with force plate measurements during the IMTP testing in physically active adults.

## 2. Materials and Methods

### 2.1. Design

We conducted an observational repeated-measures method-comparison pilot study aimed at evaluating within-session reliability and inter-device agreement during the IMTP test from April to May 2026 at the Athan Boxing Club in Concepción, Chile. This study was designed following the STROBE guidelines for observational studies [16]. This study is part of a larger project previously approved by the Ethics Committee of the Universidad Central de Chile under two protocols (Protocol code: 89/2025; Approval date: 23 September 2025) and (Protocol code: 106/2025; Approval date: 30 October 2025), and subsequently updated under (Protocol code: 35/2026; Approval date: 4 May 2026). Everyone who participated signed a written consent form in line with the Declaration of Helsinki [17].

### 2.2. Context

Muscle strength assessment presents methodological and measurement challenges, partly attributable to the high costs and limited accessibility of laboratory-grade equipment [18]. The G-Force system consisted of a custom-built force plate and an external S-type load cell configured for simultaneous force acquisition during IMTP testing. The force plate system had been previously evaluated for isometric force assessment against a reference force plate [19]. Additionally, the S-type load cell system was calibrated through internal calibration procedures prior to testing to ensure measurement consistency [20].

### 2.3. Participants

The sample for our study included 18 physically active adults (16 men and 2 women) with an average age of 27.61 ± 8.37 years, all recruited from the Athan Boxing Club using convenience sampling. Each participant gave their written informed consent before taking part in the study. We defined physical activity status following the World Health Organization’s (WHO) guidelines [21]. We confirmed eligibility with a structured questionnaire, including only those who met the criteria. Participants were recreationally trained and regularly engaged in combat sports and resistance-training activities. Additionally, all participants were free from musculoskeletal injuries at the time of testing and had previous experience performing maximal-effort strength assessments, including familiarity with the IMTP testing procedure. An a priori sample size calculation was conducted; however, the final sample size was constrained by the limited availability of participants.

The inclusion criteria were adults aged 18 to 44 years who were physically active and capable of performing maximal voluntary contractions safely, abstaining from moderate or vigorous physical activity during the 48 h prior to each testing session to avoid interference from acute exercise responses, and able to read, understand, and sign the informed consent before participation. Exclusion criteria included a diagnosis of disabling musculoskeletal disorders or non-communicable chronic diseases (e.g., hypertension, diabetes, or other cardiovascular/metabolic conditions) that contraindicated maximal effort, use of medications that could affect muscular, neuromuscular, or cardiovascular function and potentially alter force generation or responses to maximal voluntary contractions, and inability to correctly perform the physical test according to the protocol instructions.

### 2.4. Instruments

#### Signal Acquisition, Processing, and Analysis Parameters

Force measurements were obtained simultaneously using two independent systems: (i) a low-cost force plate (G-Force Alpha system; 50 × 40 cm) and (ii) an external S-type load cell mounted in series with the IMTP bar [19,20]. The load cell signal was acquired via Modbus RTU over RS-485 communication protocol, while the force plate consisted of two independent plates for the left (FL) and right (FR) feet, with total vertical ground reaction force (GRF) calculated as FL(t) + FR(t). An overview of the acquisition, processing, and analysis parameters applied to both systems is provided in Table 1.

**Figure 1:**
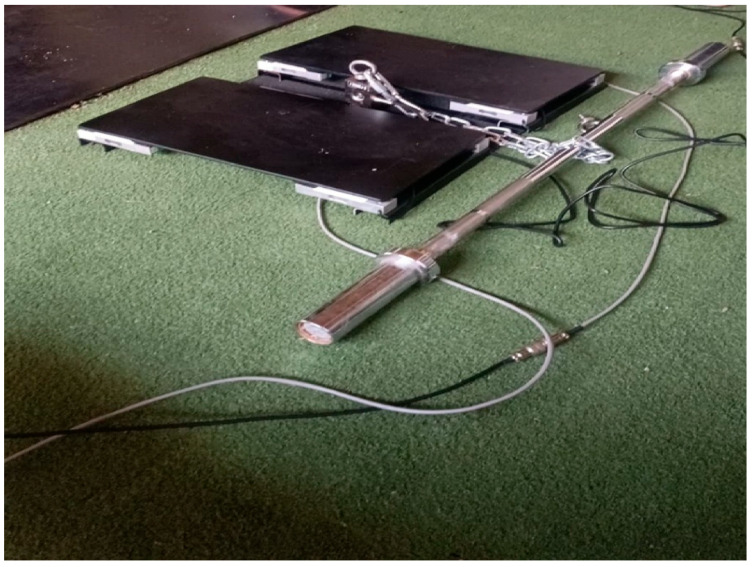
Experimental setup for the IMTP, showing the dual force plate (FL and FR) and the external S-type load cell connected in series with a chain and a fixed bar (8.5 kg). This configuration enabled simultaneous acquisition of vertical ground reaction force and tensile force during the same isometric contraction.

Figure 1 illustrates the experimental setup used during IMTP assessment. Participants stood barefoot on the dual force plate with standardized foot positioning, while the chain length was individually adjusted to position the fixed bar at mid-thigh level, thereby standardizing joint positioning across trials. During testing, participants exerted a maximal isometric pull against the immovable bar, enabling simultaneous acquisition of vertical ground reaction force and tensile force during the same contraction.

Both instruments were operated using independent acquisition systems running identical custom Python (64-bit) software to avoid resource contention and ensure consistent processing pipelines. Force signals were sampled at 1000 Hz (Δt = 1 ms), providing sufficient temporal resolution to capture rapid IMTP force transients and enabling moving-window RFD calculations. This filtering approach was applied consistently to both systems to ensure methodological symmetry in agreement analyses. The selected cutoff frequency was chosen to reduce high-frequency noise while maintaining signal stability for derivative-based outcomes. Although low-pass filtering can attenuate high-frequency components, previous IMTP and isometric force-time studies have commonly applied cutoffs within the 10–20 Hz range when the primary objective is improving signal-to-noise ratio and ensuring robust RFD estimation rather than preserving raw signal variability [22]. In addition, Butterworth low-pass filter design principles support the use of defined cutoff frequencies to improve signal-to-noise ratio and stability in processed signals, particularly in biomechanical and engineering applications [23].

Importantly, the use of a fourth-order Butterworth filter implemented with zero-phase (filtfilt) results in an effective eighth-order response; however, this does not imply inappropriate attenuation of relevant IMTP signal content, as the chosen cutoff frequency (20 Hz) lies within the commonly reported bandwidth for maximal isometric force signals. In this context, the filtering strategy prioritizes reduction in high-frequency noise while preserving the low-frequency force-time structure that predominantly drives peak force and RFD outcomes in IMTP testing (Table 1).

Computational processing was implemented in Python using NumPy, SciPy, and pandas. Computationally intensive routines were accelerated using Numba with just-in-time compilation (@jit(nopython = True, cache = True, fastmath = True)). To account for differences in signal interpretation between systems, both raw and corrected force values were computed (see Supplementary Material S1 for detailed equations).

### 2.5. Variables

#### 2.5.1. Sociodemographic and Anthropometric

Participants reported their age through a questionnaire. We measured their height using a portable stadiometer (Cescorf, São Paulo, Brazil; max height 200 cm) under strict conditions. We also looked at their height and body weight using standardized methods, which follow the guidelines set by the International Society for the Advancement of Kinanthropometry (ISAK) in terms of measurement ranges and accuracy [24].

To assess body weight, a calibrated force plate was used [19]. Body mass was calculated by dividing the measured body weight (N) by gravitational acceleration (9.81 m/s^2^). For subsequent analyses, the constant mass of the bar and chain system (8.5 kg; 83.4 N) was added to the participant’s body weight to determine the total system load.

We calculated the body mass index (BMI) as body mass in kilograms divided by height in meters squared [25]. These steps helped ensure consistency and a rigorous approach to sample characterization.

#### 2.5.2. Muscle Strength

The following variables were analyzed:

- Peak force (N): The highest value of the filtered force signal during the 5 s IMTP trial, representing maximal voluntary force production [26]. Force values were obtained from both systems (load cell and force plate) and expressed as raw and body-mass/bar-corrected values to allow inter-instrument comparison. A detailed description of signal processing, force decomposition, and correction procedures is provided in the Supplementary Material.
- RFD max (N/s): The maximum rate of force development derived from a 20 ms moving window applied to the filtered force-time curve) [26,27]. This method computes the mean slope over overlapping 20 ms intervals (1 ms step), and the highest value across the entire contraction was extracted as the RFD max, reducing noise sensitivity and improving reliability compared to instantaneous differentiation. Full mathematical formulation and implementation details are provided in the Supplementary Material.

The use of a 20 ms moving window is consistent with previous biomechanical and strength and conditioning research reporting moving-average or short epoch-based RFD calculations as a strategy to improve signal stability and reduce the influence of noise and onset detection error in rapid isometric contractions. In particular, short-window RFD approaches have been proposed as a practical alternative when onset-dependent time-bands (e.g., 0–50 ms, 0–100 ms) are sensitive to temporal misalignment between systems [28,29].

Because the load cell and force plate measure different mechanical quantities at movement initiation, systematic differences arise in force onset detection [30]. The load cell detects tensile force from a near-zero baseline, whereas the force plate records total ground reaction force, including body weight and postural adjustments. As a result, even minimal temporal misalignments (on the order of milliseconds) can lead to substantial discrepancies in early-phase RFD metrics (e.g., 0–50 ms, 0–100 ms), reflecting methodological artifacts rather than true physiological differences.

In the present study, the exclusion of onset-dependent RFD variables was primarily driven by limitations in achieving consistent and comparable onset detection across the two measurement systems under simultaneous acquisition conditions. This represents a methodological constraint inherent to inter-device comparison in IMTP testing rather than a limitation of participant performance.

Therefore, onset-dependent RFD variables were not analyzed, and only the maximum RFD derived from the moving-window method was reported.

### 2.6. Exercise Protocol

#### 2.6.1. Familiarization

To assess inter-device comparability under isometric conditions, participants did an isometric pulling task similar to the isometric mid-thigh pull [31]. The test involved pulling against a non-stretch chain connected to an S-type load cell (500 kg), standing on a G-Force Alpha force plate (50 × 40 cm) to maintain a stable position without any movement [19,20].

We standardized the joint positions to a mid-thigh pull setup, with a bit of flex at the hips and knees. Participants were asked to pull symmetrically with both hands. We recorded their individual positioning and replicated it for subsequent trials to reduce measurement errors due to posture.

During the familiarization phase, we determined body weight using the force plate before testing, as is typical in IMTP protocols [32]. An extra 8.5 kg was added to account for the weight of the barbell, which helped standardize the total load each participant had to handle. This method promotes consistency in the external load used during testing and aligns with force–time analysis methods that factor in total system weight (body mass plus any external load) for measuring force production [33].

A fixed nominal bar mass was used instead of performing trial-by-trial dynamic calibration of the full system (bar + attachments) for each participant, as the primary objective of the study was inter-device agreement under identical and standardized testing conditions rather than absolute force quantification. This approach is commonly adopted in applied IMTP research to ensure methodological consistency across trials while minimizing additional sources of variability introduced by repeated system re-calibration procedures. Importantly, this assumption introduces a constant systematic offset that is consistent across both measurement systems and therefore does not affect comparative analyses of agreement between devices.

Then, participants completed two submaximal familiarization trials (50–80% of what they thought their maximum effort would be) [32,33]. The familiarization procedure was performed immediately before data collection, following the standardized warm-up protocol. This approach is consistent with a previous IMTP study conducted in physically active participants, in which familiarization trials were incorporated within the same testing session prior to maximal data acquisition [26].

#### 2.6.2. Isometric Mid-Thigh Pulls

Each participant completed three maximal trials, with each lasting 5 s. Participants stood barefoot on the dual force plate with standardized foot positioning. The chain length was individually adjusted to position the fixed bar at the mid-thigh level for each participant, thereby standardizing joint positioning across trials. Before each attempt, participants were instructed to maintain an upright posture, grip the bar firmly, and avoid countermovement prior to force generation. They were instructed to generate force as quickly as possible and keep pushing at their maximum throughout the contraction. Standard verbal instructions were given (“pull as hard and as fast as you can, until I say stop”), which were preceded by a countdown (“3, 2, 1, pull”). Participants received consistent verbal support during each attempt.

**Figure 2:**
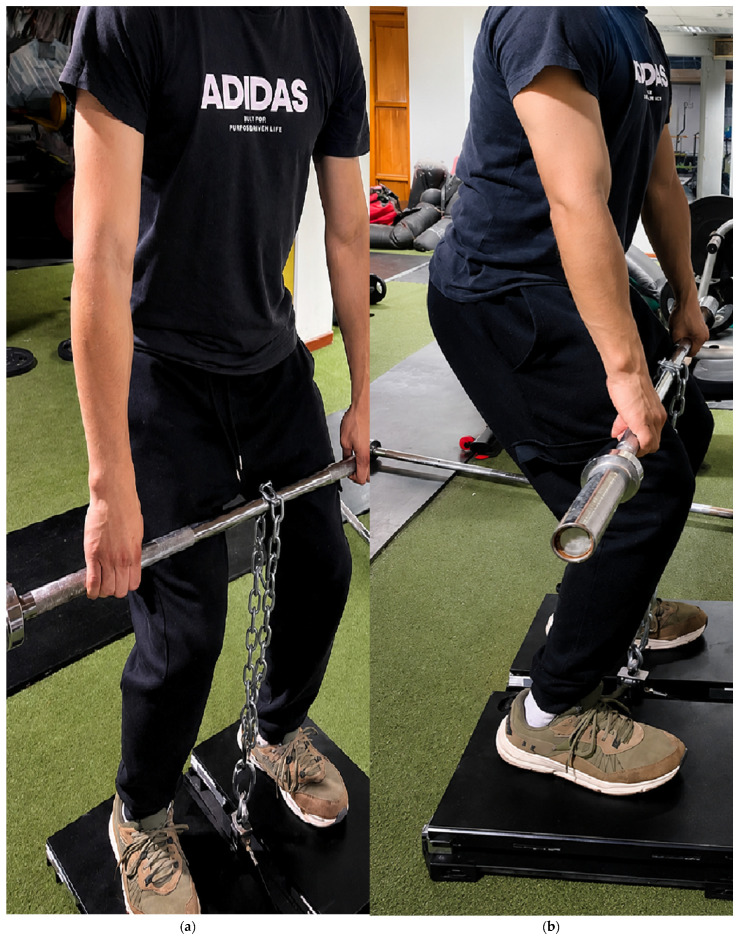
(**a**) Execution of the IMTP from the frontal view (frontal plane); (**b**) Execution of the IMTP from the sagittal view (lateral view).

Any trials that did not meet the required criteria (like visible countermovement, losing grip, or not pushing hard enough) were discarded and had to be repeated. They were allowed to rest for 2–3 min between trials [11]. Because maximal voluntary efforts may vary between attempts, multiple trials were performed to improve measurement stability and reduce the influence of isolated performance fluctuations. Trial-to-trial consistency was subsequently examined through within-session reliability analyses. For the primary statistical analysis, the average performance across the three maximal trials was used to reduce random within-subject variability and provide a more stable representation of force output during IMTP testing (see Figure 2).

#### 2.6.3. Standardization of External Load (Bar Mass)

To ensure consistency across all trials and participants, a nominal external bar mass of 8.5 kg (83.39 N) was considered during force signal interpretation. Because the force plate measured total vertical ground reaction force, including body weight and external system load, whereas the load cell measured tensile force directly applied to the bar, both raw and corrected force outputs were computed to facilitate inter-system comparison.

The same nominal bar mass correction was applied systematically across all participants and trials in order to minimize variability associated with session-specific setup differences and maintain consistency in external load treatment between systems. However, given the distinct mechanical characteristics and force transmission pathways of the force plate and load cell systems, small differences in baseline correction and force decomposition may have contributed to the fixed bias observed in peak force outcomes.

Because the external load correction represents a constant offset in the force signal, it was not expected to substantially influence derivative-based variables such as the RFD, which depends on the change in force over time rather than absolute force values [34]. Force onset was defined as a 5 N threshold above baseline and was used for signal alignment purposes only [35,36]. This low absolute threshold was selected to minimize the influence of baseline noise while ensuring consistent detection of the initiation of force production, in line with previous IMTP methodological recommendations regarding onset identification [35,36].

### 2.7. Bias

Several methodological limitations should be considered when interpreting the present findings:

- Sample representativeness limitation: We only tested young, physically active adults, which may limit the external generalizability of the findings to broader populations such as older adults, elite athletes, or individuals with neuromuscular or clinical conditions [37]. Although the systems were designed to measure the same physical construct (force), additional studies across different neuromuscular profiles would strengthen the applicability of these findings in other populations.
- Within-session performance variability: Even though we included practice trials and standard rest periods, it is possible that learning effects or fatigue from multiple trials in one session could still influence performance [38,39]. Because both systems recorded each contraction simultaneously, these effects were expected to influence both devices concurrently rather than systematically favor one system over the other. However, potential differences in signal sensitivity or response characteristics between devices under varying contraction conditions cannot be completely excluded.
- Measurement and signal processing bias: We calibrated both systems and used standard filtering methods, but the early rate of force development might still be sensitive to slight variations in sensor performance and signal quality, which could affect comparisons between systems [40].
- Inter-system comparison limitation: We compared two measurement systems against each other, not against an external gold-standard force plate. So the findings reflect how well the devices agree with each other, rather than indicating absolute validity [41]. Although both systems had been previously evaluated independently, validity may vary according to testing configuration, synchronization procedures, signal processing strategies, and outcome selection. Therefore, the present study should be interpreted primarily as an assessment of concurrent inter-device agreement within this specific simultaneous IMTP configuration rather than as a criterion validity investigation. Furthermore, while systematic differences were observed between systems, these findings do not necessarily indicate overestimation or underestimation by either device, as no external criterion reference was used under identical testing conditions. Differences in force transmission pathways and signal interpretation between systems may also have contributed to the observed fixed bias.
- Design limitation: Since this was a cross-sectional and observational study, we could not completely control confounding variables, and we cannot draw causal conclusions about the differences between systems [42].

### 2.8. Sample Size

For this pilot study, we set the sample size based on its exploratory nature and some reliability research recommendations [43]. In studies focused on inter-device agreement (e.g., ICC and Bland–Altman analyses), sample sizes between 20 and 30 participants are commonly considered acceptable for providing stable estimates of reliability and limits of agreement in applied biomechanics contexts [39,40,41].

Methodological studies suggest that having at least 15 to 20 participants is ideal for getting stable estimates and narrow confidence intervals. Previous work on the IMTP has often had smaller sample sizes, usually around 13 to 19 participants, when looking at biomechanical reliability and inter-system agreement [34,44,45]; However, these studies vary substantially in analytical approach and should be interpreted as contextual rather than prescriptive benchmarks for agreement-based designs.

Although an a priori power analysis was conducted using G*Power (version 3.1.9.7) for exploratory methodological planning purposes, the primary aim of the study was not hypothesis testing but inter-device agreement assessment. Therefore, the power analysis should be interpreted cautiously and not as the sole determinant of sample adequacy for reliability statistics.

Assuming a moderate effect size (f = 0.25), a significance level of α = 0.05, statistical power of 0.95, three repeated measurements, and an assumed correlation among repeated measures of 0.85, the analysis indicated a minimum required sample size of 14 participants.

Convenience sampling was employed, recruiting participants from nearby gyms. This study took an exploratory approach, focusing on comparing the systems rather than testing a specific hypothesis. While larger samples are generally recommended to improve precision in agreement studies, the final sample of 18 participants exceeded the minimum estimate derived from the exploratory power analysis and falls within the range commonly reported in similar IMTP reliability literature.

### 2.9. Statistical Analysis

We analyzed the data using IBM SPSS Statistics version 27.0 (IBM Corp., Armonk, NY, USA). First, we checked for normality using the Shapiro–Wilk test and assessed homogeneity of variances with Levene’s test. Descriptive statistics were presented as means and standard deviations.

All inferential statistical analyses were performed using JASP version 0.96.4 (JASP Team, University of Amsterdam, The Netherlands). Paired sample *t*-tests were used to assess systematic differences between the force plate and load cell system, and Cohen’s d was calculated to quantify effect sizes. Effect sizes were interpreted as trivial (<0.25), small (0.25–0.50), moderate (0.51–1.00), and large (>1.00) [46].

Intraclass correlation coefficients (ICC) and standard error of measurement (SEM) were obtained using JASP version 0.96.4 (JASP Team, University of Amsterdam, The Netherlands), applying a two-way mixed effects model. For all ICC analyses, a single configuration was used. Given the agreement-focused nature of the study, ICC results were interpreted as measures of relative agreement between systems rather than test–retest reliability alone. For ICC analyses, single-measure ICC (3,1) was prioritized for primary interpretation to reflect individual trial applicability in applied settings, while average-measure ICC (3,k) was used as a complementary descriptor of trial-averaged performance consistency [47].

For each participant, three maximal trials were recorded. Although all three trials were included to characterize within-session performance stability, primary agreement analyses were additionally supported by trial-averaged values to reduce random within-subject variability while maintaining comparability with previous IMTP literature.

Absolute reliability for each system was assessed using the coefficient of variation (CV%), which was calculated manually as the standard deviation divided by the mean, multiplied by 100 to express it as a percentage. Additionally, CV values were interpreted as indicators of within-system relative variability rather than sole markers of agreement between systems. Although log-transformed typical error (TE) has been recommended in some sports science contexts as a more robust estimator of absolute reliability under heteroscedastic conditions, the CV% was retained in the present study to ensure comparability with previous IMTP and isometric strength literature, where CV-based metrics remain the most commonly reported index of within-system variability. The minimal detectable change (MDC) was also calculated manually as 1.96 × √2 × SEM [41,48,49].

Bland–Altman analyses, including mean bias and 95% limits of agreement, were performed using GraphPad Prism version 8.4.2 (GraphPad Software, San Diego, CA, USA) [50]. SEM and MDC were not calculated for inter-system agreement analyses, as these metrics are specific to within-system measurement error. Instead, agreement was interpreted using ICC values together with Bland–Altman statistics.

To further assess the presence of proportional bias, linear regression analyses were performed following the Bland–Altman approach, with the difference between systems (force plate − load cell) entered as the dependent variable and the mean of both systems as the independent variable [51,52]. This analysis was conducted separately for each outcome (peak force and RFD max) using JASP. The presence of proportional bias was determined based on the statistical significance of the regression slope (β coefficient), with non-significant slopes indicating absence of proportional bias and suggesting that differences between systems were constant across the range of measurements [53]. Regression coefficients (unstandardized β), 95% confidence intervals, and *p*-values were reported. Proportional bias was interpreted as evidence of systematic difference varying across the measurement range when statistically significant slopes were observed. Finally, statistical significance was set at *p* < 0.05 (two-tailed).

## 3. Results

Table 2 provides a snapshot of the sample’s sociodemographic features. The participants had an average age of 27.61 ± 8.37 years, weighed in at about (82.72 ± 18.86 kg), and stood around (172.06 ± 6.69 cm), leading to a body mass index (BMI) of 27.81 ± 5.26 kg/m^2^.

In Table 3, intra-session reliability for both systems is presented across three IMTP trials. Peak force showed excellent relative reliability in both devices, with comparable ICC values for the load cell system (ICC = 0.979) and the G-Force plate (ICC = 0.976). However, ICC values should be interpreted alongside absolute and within-subject variability metrics, as they primarily reflect relative ranking consistency rather than absolute agreement. Absolute variability was similar between systems, ranging from 32.3% to 33.7%. Measurement error was slightly higher for the force plate (SEM = 200.4 N; MDC = 555.7 N) compared to the load cell system (SEM = 185.6 N; MDC = 514.5 N).

RFD max showed lower reliability in both systems, particularly in the force plate (ICC = 0.655), with high variability (CV = 63.1%) and large measurement error (SEM = 6338 N/s; MDC = 17,561.9 N/s). The load cell system showed slightly higher reliability for RFD (ICC = 0.734), although variability remained high (CV = 59.7%) with substantial measurement error (SEM = 6572 N/s; MDC = 18,216.7 N/s). These CV values indicate substantial within-session variability for RFD outcomes in both systems, suggesting limited absolute stability and reduced practical sensitivity of RFD max as a repeatable metric under the present IMTP testing conditions. These results indicate limited within-session stability for RFD outcomes across both systems.

**Figure 3:**
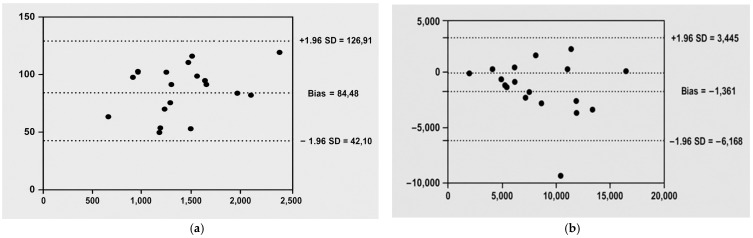
(**a**) Bland–Altman plots showing agreement between the force plate and load cell system for Peak Force (N) in IMPT (N); (**b**) Bland–Altman plots showing agreement between the force plate and load cell system for RFD max (N/s) in IMPT.

Table 4 and Figure 3 illustrate the agreement between the force plate and load cell system. For peak force, the agreement was excellent (ICC = 0.999; 95% CI: 0.998 to 1.000), with a positive systematic bias (84.48 N), indicating that the force plate generally yielded higher values. The 95% limits of agreement ranged from 42.10 to 126.90 N, reflecting relatively narrow dispersion of differences between systems. A statistically significant difference was observed for peak force (*p* < 0.001). Although the effect size was large (d = 3.90), the absolute bias between devices was relatively small (84.48 N; ~6% of mean peak force). Therefore, the large, standardized effect size likely reflects the low dispersion of the inter-system differences rather than a substantial absolute discrepancy, despite the very high relative agreement observed.

In contrast, RFD max demonstrated lower agreement (ICC = 0.887; 95% CI: 0.699 to 0.958) and greater variability between systems. It showed a negative bias (−1361 N/s), indicating that higher values were generally obtained from the load cell system. The limits of agreement were wide (−6168 to 3445 N/s), reflecting substantial absolute disagreement between systems in early-phase force-time characteristics. A statistically significant difference for RFD max was also observed (*p* = 0.031), with a moderate effect size (d = 0.55), suggesting meaningful differences in absolute values between systems. Importantly, ICC values primarily reflect relative consistency in the ranking of participants across devices, whereas Bland–Altman analysis and effect sizes provide complementary information regarding absolute agreement and systematic differences between measurement systems. In this context, the present results indicate that, although relative agreement between systems is high, notable absolute differences persist, particularly for peak force and RFD outcomes.

To further explore the nature of the agreement observed in the Bland–Altman analysis, linear regression models were conducted to assess the presence of proportional bias between systems. For peak force, no significant association was found between the differences and the mean values (β = 0.017, 95% CI: −0.008 to 0.043, *p* = 0.159), indicating that the observed bias was constant across the measurement range, consistent with the fixed bias identified in the Bland–Altman plot. Similarly, for RFD max, no significant relationship was observed (β = −0.185, 95% CI: −0.527 to 0.157, *p* = 0.268), suggesting that the variability and wider limits of agreement were not dependent on the magnitude of force production. These findings confirm that, despite differences in absolute agreement, particularly for RFD max, the discrepancies between systems were not influenced by increasing force levels (Table 5).

## 4. Discussion

This study aimed to compare the agreement and reliability of the G-Force system (force plate and load cell) for the mid-thigh isometric pull test in physically active adults. A key finding was that the level of agreement between systems depended on the specific force-time variable analyzed, with excellent agreement observed for peak force and lower agreement for RFD-related metrics.

For peak force, the systems demonstrated excellent agreement (ICC = 0.999), with narrow limits of agreement and a statistically significant systematic difference. While the standardized effect size was large (d = 3.90), this result should be interpreted in the context of the low variability of the inter-system differences, as the absolute bias was 84.48 N (approximately 6% of the mean peak force). Collectively, these findings indicate a consistent systematic difference between devices despite their excellent relative agreement. Importantly, this effect size indicates a large and practically meaningful systematic bias between devices, reflecting consistent differences in absolute force values rather than a trivial discrepancy. Despite this, the direction and magnitude of the bias remained stable across the measurement range, as confirmed by the absence of proportional bias (β = 0.017; 95% CI: −0.008 to 0.043; *p* = 0.159). These findings reinforce previous evidence indicating that peak force is a robust and highly reproducible metric across different force measurement technologies when standardized testing and signal processing procedures are applied [31,41]. Importantly, peak force appears less sensitive to methodological variation compared to derivative-based variables, supporting its use as a primary outcome in both laboratory and field-based strength assessments [54].

In contrast, RFD max showed lower agreement between systems (ICC = 0.887), wider limits of agreement, and a moderate effect size (d = 0.55), indicating meaningful systematic and random differences between devices. Regression analysis also showed no proportional bias (β = −0.185; 95% CI: −0.527 to 0.157; *p* = 0.268), suggesting that inter-system differences were consistent across the range of RFD values. This aligns with previous literature highlighting the high methodological sensitivity of RFD, particularly to signal processing choices such as filtering, onset detection, and time-window selection [55,56]. Even when identical processing pipelines are applied, small differences in sensor dynamics and sampling characteristics can be amplified in derivative-based variables, leading to reduced cross-device comparability [57].

The lower inter-system agreement observed for RFD max should be interpreted in the context of the inherent methodological sensitivity of early-phase force-time variables [32,35]. Unlike peak force, RFD is highly dependent on signal processing choices, including filtering, time-window selection, and temporal alignment of the force signal [28,34]. In the present study, both systems were processed using identical filtering and analysis pipelines; however, even minor differences in sensor characteristics and signal timing can produce amplified discrepancies in derivative-based metrics [34]. Additionally, the use of a 20 ms moving window, while reducing noise compared to instantaneous differentiation, does not fully eliminate sensitivity to high-frequency fluctuations and baseline instability [22,32]. Therefore, the observed disagreement likely reflects the combined effect of measurement system characteristics and the intrinsic instability of RFD as a derived variable rather than a limitation of either device alone [34].

These findings are consistent with the intra-session reliability results obtained in the present study. Peak force exhibited excellent relative reliability in both systems (ICC = 0.976–0.979) with moderate coefficients of variation (32.3–33.7%), whereas RFD max showed substantially lower reliability (ICC = 0.655–0.734) and markedly higher variability (CV = 59.7–63.1%). This pattern reinforces the notion that peak force is a stable performance metric, while RFD is inherently more variable and method-sensitive, particularly in non-isometric or rapid force transitions [22,28,44].

From a methodological perspective, even though both systems were synchronized and processed using identical filtering procedures, small differences in sensor architecture likely influenced RFD estimation. This is especially relevant in early-phase force analysis, where short time windows amplify the impact of baseline noise and transient fluctuations [29,58]. Consequently, agreement between systems appears to be more dependent on the derived nature of the variable than on the underlying force signal itself.

Regarding reliability interpretation, ICC should be understood as a measure of relative consistency rather than absolute agreement. Therefore, the very high ICC observed for peak force reflects strong preservation of inter-individual ranking between systems rather than exact equivalence of values. Importantly, the associated confidence intervals in Table 4 (0.998–1.000 for peak force and 0.699–0.958 for RFD) now show adequate precision and no anomalous widening, supporting the stability of the estimates in this sample. Nevertheless, ICC should always be interpreted alongside Bland–Altman analysis to fully characterize both systematic bias and absolute agreement [51,52,53,59,60].

From a practical standpoint, these results support high relative comparability but do not support full interchangeability of both systems for peak force assessment in applied settings, particularly when standardized protocols are used. The presence of a large systematic bias indicates that, although devices track changes in a similar way, absolute values are not directly interchangeable without calibration or correction factors. However, caution is warranted when interpreting RFD-derived outcomes, as both intra- and inter-system evidence indicate higher variability and lower agreement. This distinction is critical for practitioners and researchers when selecting force-time variables for monitoring strength and neuromuscular performance [56,60].

Finally, while the force plate demonstrated strong agreement with the load cell system for peak force, these findings should be interpreted as inter-system agreement rather than criterion validity, given the absence of a true gold standard reference system in this context. Therefore, the present results contribute to the growing evidence supporting the use of low-cost force measurement systems, while also highlighting their limitations for derivative-based variables such as RFD. Additionally, the generalizability of these findings should be interpreted with caution due to the marked sex imbalance in the study sample, which consisted predominantly of men (16 men and 2 women). As sex-related differences in force production characteristics and neuromuscular performance may influence force-time outcomes, future studies including more balanced and representative samples are warranted to confirm the applicability of these findings across both sexes.

## 5. Conclusions

The G-Force system demonstrated strong agreement with a load cell system for the assessment of peak force during the IMTP, suggesting its potential as a cost-effective option for practical applications. However, agreement between the two systems for RFD was less consistent and showed greater variability, indicating that they may not be interchangeable for early-phase force–time measurements. Overall, these findings suggest that measurement agreement is dependent on the specific variable being assessed. The present findings provide strong support for peak force as a robust and reproducible metric. Conversely, RFD appears to be more sensitive to methodological variations and exhibits lower consistency across measurement systems, warranting greater caution in its interpretation. Moreover, despite the high relative agreement observed for peak force, the presence of a substantial systematic bias suggests that absolute values obtained from the two systems should not be considered directly interchangeable without appropriate adjustment procedures.

## Figures and Tables

**Table 1:** Signal acquisition, processing, and analysis parameters applied.

Category	Parameter	Configuration
Measurement systems	Instruments	S-type load cell (Modbus RTU/RS-485) and dual force plate (FL + FR)
	Synchronization	Simultaneous acquisition using two independent computers (one per system)
Acquisition	Sampling frequency	1000 Hz (Δt = 1 ms)
	Software	Python 3.13 (64-bit); NumPy, SciPy, pandas; Numba (JIT)
Calibration	Load cell	Chronojump-based protocol with known loads
	Force plate	Static in situ calibration with known loads
Signal processing	Filter type	Butterworth low-pass (IIR)
	Order	4 (effective order 8 via filtfilt)
	Cutoff frequency	20 Hz
	Phase correction	Zero-phase filtering (filtfilt)
	Resampling	Linear interpolation to uniform 1000 Hz grid
	Force onset	Threshold of 5 N above baseline
Peak force analysis	Outputs	Load cell (raw/net) + Force plate (raw/net)
RFD max analysis	Method	20 ms moving window (1 ms step)
	Output	Maximum RFD (N/s), no mass correction
Constants	Gravity	g = 9.81 m·s^−2^
	External load	Bar mass = 8.5 kg (83.39 N)
Excluded variables	Onset	Onset-dependent metrics (early/late RFD, time-to-peak)

**Table 2:** Sociodemographic characteristics of the sample analyzed (*n* = 18).

Variables	X ± SD	95% CI
Age (years)	27.61 ± 8.37	23.45 to 31.78
Weight (kg)	82.72 ± 18.86	73.34 to 92.10
Height (cm)	172.06 ± 6.69	168.73 to 175.38
BMI (kg/m^2^)	27.81 ± 5.26	25.19 to 30.42

X: mean, SD: standard deviation, CI: confidence interval.

**Table 3:** Intra-session reliability of the force plate and load cell system during three IMTP trials (*n* = 18).

Instruments	Variable	Trial 1 (X ± SD)	Trial 2 (X ± SD)	Trial 3 (X ± SD)	SEM	MDC	CV (%)	ICC (95% CI)
Force plate	Peak Force (N)	1336.48 ± 397.76	1397.27 ± 450.16	1377.34 ± 475.89	200.4	555.7	32.3	0.976 (0.948 to 0.990)
	RFD max (N/s)	6963.78 ± 3517.13	7795.58 ± 6434.78	7219.72 ± 3191.40	6338	17,561.9	63.1	0.655 (0.219 to 0.861)
Load cell	Peak Force (N)	1243.81 ± 395.40	1315.94 ± 434.41	1297.92 ± 468.17	185.6	514.5	33.7	0.979 (0.953 to 0.991)
	RFD max (N/s)	8135.82 ± 4104	9157.65 ± 6363.42	8769.76 ± 4845.06	6572	18,216.7	59.7	0.734 (0.415 to 0.892)

X: mean; SD: standard deviation; ICC: intraclass correlation coefficient; CI: confidence interval; SEM: standard error of measurement; MDC: minimal detectable change; CV: coefficient of variation.

**Table 4:** Between-system agreement between the force plate and load cell system during the IMTP test (*n* = 18).

Variable	Force Plate (X ± DS)	Load Cell (X ± SD)	ICC (95% CI)	*p*-Value	d
Peak Force (N)	1370.37 ± 432.26	1285.89 ± 424.76	0.999 (0.998 to 1.00)	<0.001	3.90
RFD max (N/s)	7326.36 ± 3520.45	8687.74 ± 4164.12	0.887 (0.699 to 0.958)	0.031	0.55

X: mean; SD: standard deviation; ICC: intraclass correlation coefficient; CI: confidence interval.

**Table 5:** Linear regression analysis for proportional bias between the force plate and load cell system during the IMTP test.

Outcome (Difference)	Predictor (Mean)	Beta (Unstandardized)	CI (95%)	*p*-Value
Peak Force (N)	Peak Force	0.017	−0.008 to 0.043	0.159
RFD max (N/s)	RFD max	−0.185	−0.527 to 0.157	0.268

## Data Availability

The data from this article will be made available by the author’s reasonable request.

## Supplementary Materials

The following supporting information can be downloaded at: https://www.mdpi.com/article/10.3390/s26134178/s1. Supplementary Material S1: Signal Processing, Force Computation, and RFD Analysis Procedures.
