# Supplementary material for: Agreement and Reliability of the G-Force System: Force Plate and Load Cell for the Isometric Mid-Thigh Pull in Physically Active Adults: A Repeated-Measures Method-Comparison Study
Source: Sensors (Basel). 2026 Jul 2;26(13):4178. doi: 10.3390/s26134178 (PMC13363757; doi:10.3390/s26134178)
Supplement: Supplementary file 1 [file sensors-26-04178-s001.zip › sensors-4316088-supplementary.pdf]

Peak Force (N)

Peak force (PF, or F_{max}) was defined as the maximum value of the filtered force–time signal within the analysis window:

$$F_{\text{max}} = \max\{\tilde{F}(t): t \in [t_0, t_N]\}$$

where $\tilde{F}(t)$represents the force signal after low-pass filtering using a zero-phase Butterworth filter.

To allow transparent comparison between measurement systems, four peak force values were computed for each trial:

- Load cell (reported): Maximum tensile force recorded by the load cell.
- Load cell (adjusted): Load cell signal corrected for the external load (bar mass).
- Force platform (raw): Maximum vertical ground reaction force (GRF), including body weight and external load.
- Force platform (net): GRF corrected to isolate the active force generated by the participant.

The corrected values were computed as follows:

$$F_{\text{cell, adj}} = F_{\text{cell, reported}} - F_{\text{bar}}$$
$$F_{\text{platform, net}} = F_{\text{platform, raw}} - m_{\text{subject}} \cdot g - F_{\text{bar}}$$

where

- m_{subject}: body mass
- $g = 9.81$ m\cdotps^{-2}
- $F_{\text{bar}} = 83.39$ N, corresponding to a bar mass of 8.5 kg.

The load cell measures tensile force transmitted to the bar and does not include body weight. Therefore, only the external load was removed from this signal. In contrast, the force platform measures total GRF, requiring subtraction of both body weight and external load to obtain net force.

The force platform net and load cell adjusted values were considered the most appropriate for inter-instrument comparison, as both represent the active force applied to the system under equivalent mechanical assumptions.

Python Implementation (Peak Force)

Peak force was computed directly from the filtered signal sampled at 1000 Hz using vectorized NumPy operations:

```
# F̃(t) = force signal filtered with zero-phase Butterworth filter (variable f_new)

peak_force_raw = float(np.max(f_new))   # Raw peak force (N)

# Net values (post-processing):
g            = 9.81
BW           = body_mass_kg * g          # body weight (N)
BAR_N      = 83.39                       # 8.5 kg · 9.81 m/s²

loadcell_net = loadcell_raw - BW - BAR_N
platform_net = platform_raw - BW - BAR_N
```

By construction, the difference between the force platform and the load cell is numerically identical whether calculated using raw values (platform raw − load cell reported) or net values (platform net − load cell net), since the same constant is subtracted from both sides of the equation. This property confirms that the offset between instruments does not depend on body weight or bar mass, and that any observed systematic difference is attributable to transducer calibration, signal filtering, or the tare criteria applied by each system.

Rate of Force Development (RFD, N·s^{-1})

The rate of force development (RFD) was defined as the slope of the force–time curve:

$$RFD(t) = \frac{dF}{dt}$$

Because instantaneous derivatives computed at high sampling rates are sensitive to noise, a moving-window approach was used.

RFD Computation Using a 20 ms Moving Window

Let $\tilde{F}(t)$be the filtered force signal sampled at 1000 Hz. A moving window of width $W = 20$ mswas applied. For each index i, the average slope over the window was computed as:

$$RFD_i = \frac{\tilde{F}(t_i + W) - \tilde{F}(t_i)}{W}$$

where $W = 0.020$ sand corresponds to 20 samples.

The maximum RFD was defined as:

$$RFD_{\max,20ms} = \max\{RFD_i\}$$

This approach produces overlapping windows (95% overlap), improving robustness and reducing sensitivity to high-frequency noise while preserving the temporal characteristics of the signal.

Python implementation (RFD)

The full vectorized implementation, as included in the source code of the analysis software, is as follows:

```
def calculate_rfd_window_interp(t, f, window_ms, fs_interp=1000):
    """Sliding window on a fine grid: (F(t+W) - F(t)) / W"""
    t = np.asarray(t, dtype=np.float64)
    f = np.asarray(f, dtype=np.float64)
    if len(t) < 2:
        return np.array([])

    W = window_ms / 1000.0          # window in seconds (W = 0.020 s)
    if t[-1] - t[0] < W:
        return np.array([])

    dt_new = 1.0 / fs_interp        # time step of the grid (1 ms)
    t_new  = np.arange(t[0], t[-1] + 1e-12, dt_new)
    f_new  = np.interp(t_new, t, f) # signal on a uniform 1000 Hz grid

    w = int(round(W / dt_new))      # window size in samples (20)
    if w < 1 or len(t_new) - w - 1 < 0:
        return np.array([])

    # Optimized path using Numba JIT (compiled to native code)
    if NUMBA_AVAILABLE:
        return _rfd_ventana_numba(t_new, f_new, w)

    # Fallback path using pure NumPy (mathematically equivalent)
    return (f_new[w:] - f_new[:-w]) / W



# Computation of the maximum value of the sliding window:
rfd_w        = calculate_rfd_window_interp(t, f_filtered, window_ms=20)
RFD_max_20ms = float(np.max(rfd_w))   # reported value in N/s
```

This implementation computes the difference between the signal evaluated at t+W and t across the entire time series in a vectorized manner, ensuring computational efficiency and numerical consistency.

Invariance of RFD to additive constants

RFD is mathematically invariant to constant offsets in the force signal. If a constant C is added or subtracted:

$$\frac{d}{dt}(\tilde{F}(t) - C) = \frac{d\tilde{F}(t)}{dt}$$

Similarly, for the moving window:

$$\left(\tilde{F}(t + W) - C\right) - (\tilde{F}(t) - C) = \tilde{F}(t + W) - \tilde{F}(t)$$

Therefore, RFD values are unaffected by body weight, bar mass, or baseline offsets, and no correction was applied to RFD calculations for either measurement system.
